# Demonstrating Benefit-Risk Profiles of Novel Therapeutic Strategies in Kidney Transplantation: Opportunities and Challenges of Real-World Evidence

**DOI:** 10.3389/ti.2022.10329

**Published:** 2022-05-03

**Authors:** Ilkka Helanterä, Jon Snyder, Anders Åsberg, Josep Maria Cruzado, Samira Bell, Christophe Legendre, Hélio Tedesco-Silva, Giovanna Tedesco Barcelos, Yvonne Geissbühler, Luis Prieto, Jennifer B. Christian, Erik Scalfaro, Nancy A. Dreyer

**Affiliations:** ^1^ Department of Transplantation and Liver Surgery, Helsinki University Hospital and University of Helsinki, Helsinki, Finland; ^2^ Hennepin Healthcare Research Institute, Minneapolis, MN, United States; ^3^ Department of Transplantation Medicine, Oslo University Hospital-Rikshospitalet, Oslo, Norway; ^4^ Department of Pharmacy, University of Oslo, Oslo, Norway; ^5^ Department of Nephrology, Bellvitge University Hospital, L’Hospitalet de Llobregat, Barcelona, Spain; ^6^ Bellvitge Biomedical Research Institute-IDIBELL, L’Hospitalet de Llobregat, Barcelona, Spain; ^7^ Clinical Sciences Department, Faculty of Medicine and Health Sciences, University of Barcelona, Barcelona, Spain; ^8^ Division of Population Health and Genomics, School of Medicine, University of Dundee, Dundee, United Kingdom; ^9^ The Scottish Renal Registry, Scottish Health Audits, Public Health and Intelligence, Information Services, Glasgow, United Kingdom; ^10^ Hôpital Necker, Assistance Publique Hôpitaux de Paris (AP-HP) and Université Paris Descartes, Paris, France; ^11^ Nephrology Division, Hospital do Rim, Escola Paulista de Medicina, Universidade Federal de São Paulo, São Paulo, Brazil; ^12^ Novartis Pharma AG, Basel, Switzerland; ^13^ IQVIA Real-World Solutions, Durham, NC, United States; ^14^ IQVIA Real-World Solutions, Basel, Switzerland; ^15^ IQVIA Real-World Solutions, Cambridge, MA, United States

**Keywords:** data harmonization, extension studies, real-world evidence, registries, kidney transplantation

## Abstract

While great progress has been made in transplantation medicine, long-term graft failure and serious side effects still pose a challenge in kidney transplantation. Effective and safe long-term treatments are needed. Therefore, evidence of the lasting benefit-risk of novel therapies is required. Demonstrating superiority of novel therapies is unlikely via conventional randomized controlled trials, as long-term follow-up in large sample sizes pose statistical and operational challenges. Furthermore, endpoints generally accepted in short-term clinical trials need to be translated to real-world (RW) care settings, enabling robust assessments of novel treatments. Hence, there is an evidence gap that calls for innovative clinical trial designs, with RW evidence (RWE) providing an opportunity to facilitate longitudinal transplant research with timely translation to clinical practice. Nonetheless, the current RWE landscape shows considerable heterogeneity, with few registries capturing detailed data to support the establishment of new endpoints. The main recommendations by leading scientists in the field are increased collaboration between registries for data harmonization and leveraging the development of technology innovations for data sharing under high privacy standards. This will aid the development of clinically meaningful endpoints and data models, enabling future long-term research and ultimately establish optimal long-term outcomes for transplant patients.

## Introduction

While short-term survival rates of transplanted grafts and patients have improved in past decades, progress of long-term graft survival is still limited. In addition to the highly specialized surgery, long-term immunomodulatory treatment is needed to prevent rejection and allograft failure (1). The average graft half-life is around 12 years, with around one in five kidney transplant patients experiencing graft failure within the first 5 years (2, 3). Limited long-term effectiveness of immunomodulatory treatments, reduced adherence over time and long-term adverse events (AEs), calls for improvement of lasting outcomes for post-transplant patients (4).

Demonstrating superiority of novel therapies and strategies in the long-term is challenging in conventional randomized controlled trial (RCT) settings. This is due to statistical challenges presented by the requirement to demonstrate benefits with long-term follow-up and large sample sizes. Resulting in increased operational risks (e.g., costs, trial incompletion) for sponsors, they also pose a high operational burden on patients and physicians. The need for shorter term, clinically meaningful endpoints that are predictive of longer-term outcomes has been extensively described (5–7).

Whereas regulatory hurdles limit opportunities for novel therapeutics in RCTs to demonstrate improved graft and patient survival in the short-term (e.g., limitation of recognized endpoints), studies in real-world (RW) treatment settings offer new possibilities to generate evidence. With generalizable cohorts, RW settings have a broader relevance and efficiency compared to RCTs; provided that data elements relate to accurately recognized clinical phenomena and are comparable across settings (5–7).

To expand the scientific understanding of innovative evidence generation in kidney transplantation, a scientific discussion was initiated by Novartis in 2020, including a panel of leading nephrologists, scientists, transplant registry experts, and drug development professionals. Participants were invited based on clinical research in kidney transplantation and/or experience in registry and real-world data (RWD)^1^ collection. The group included representatives from identified major transplant registries interested in collaboration. This viewpoint examines the current limitations of RCTs and outlines the opportunities of employing RW evidence (RWE)^2^ to evaluate novel drug therapies in kidney transplantation (8). The viewpoint further elaborates on the systematic review of renal registries by Liu et al. in 2015 (9), by identifying the most relevant RWD sources to characterize the benefit-risk profile of novel therapeutic strategies in kidney transplantation, while making a critical assessment of the challenges that generating RWE entails.

### Current Limitations of Conventional Randomized Controlled Trials in Kidney Transplantation

Long-term data is needed to understand patient outcomes beyond the one-to-three-year time-point usually considered in RCTs. Currently there is limited follow-up data available from clinical trials for kidney transplants, particularly in later years post-transplant, partly due to the high number of complex data elements (e.g., donor and recipient characteristics, transplantation procedure, acute rejection, antibody-mediated rejection, calcineurin inhibitor nephrotoxicity, scoring of inflammation from tissue biopsies etc.) (1, 10).

One of the issues are the high discontinuation rates (15–30%) observed in the first year of many immunosuppressive drug trials. Examples of this can be found in recent immunosuppressive drug RCTs in which the main reasons for patient discontinuation were AEs, severe refractory rejection or ineligibility (11, 12).

Classical RCT settings are unlikely to fulfil needs for long-term outcome data as they require large sample sizes leading to an operational and financial burden, resulting in very few patients, physicians, and sponsors (government, commercial or academic) being willing to participate in studies that require long years of clinical follow-up (13). RCTs also typically have restrictive inclusion/exclusion criteria, which can lead to the limited generalizability of trial results.

The current standard of care (SoC) provides excellent short-term outcomes in suitable donor-recipient combinations; therefore, it is difficult to exceed SoC outcomes in RCTs of novel treatments. The currently accepted endpoints by regulatory authorities (graft survival, graft function, or biopsy-proven acute rejection) provide mostly short-term outcomes, rather than long-term results (14). There are also ethical concerns due to impaired clinical equipoise: if a treatment shows short-term superiority, and potential for long-term benefit, it might not be considered ethical to include a control arm for long-term results (15). Yet, novel treatments and therapies need to be tested with long-term treatment outcomes and patient wellbeing in mind, which is often difficult to achieve within RCT settings. Advancements in graft survival improves patient quality of life, reducing both the risk of return to dialysis and the demand for a limited donor organ supply (1).

The authors believe that studies of sub-groups (e.g., hyperimmunized, desensitized, and perfused organs etc.), and non-ideal donor-recipient combinations, could demonstrate superiority of novel treatments in situations where SoC is not yet sufficient. There is also little inclusion of non-immunological aspects of kidney transplantation that should be considered (hypertension, post-transplant diabetes, reno protective therapies, hyperparathyroidism, and urinary tract infection, etc.) (16). Higher risk populations may represent an alternative to prove superiority as event rates of interest are likely to be more frequent, required sample sizes smaller, and observation periods shorter.

There is a need to improve the relevance and inclusion of patient-centric and patient reported outcomes in future research, as outlined by the Standardised Outcomes in Nephrology (SONG) initiative (17). Few trials study quality of life and patient concerns, however, some national and international registries do collect this information (18). In conjunction with strategies for better long-term follow-up, the growing need for more consistent collection of PROs, and short-term outcomes in sub-populations, RW study designs can provide alternative approaches to interventional clinical study designs.

A common understanding on surrogate endpoints in kidney transplantation is required to improve the comparability of data as these do not directly measure clinical benefits, but rather predict the likelihood of a clinical benefit (19). Some surrogate endpoints are a small subset of biomarkers, “laboratory measurements that reflects the activity of a disease process” (20), and should stem from data routinely captured in clinical practice, deemed acceptable by health authorities, and compatible with information regularly captured in RCTs (18). However, these often require a breadth of clinical data not always captured in routine healthcare data collection and/or registries (18).

Kidney transplant biomarkers were categorized by Mannon et al. as either pre-transplant, early post-transplant and late post-transplant markers (5). One pre-transplant biomarker—the Eplet-mismatch score has been accepted into the Biomarker Qualification Program, with attempts to qualify it as a prognostic biomarker (5). The iBox, an early post-transplant biomarker, is used to predict long-term allograft failure after a fairly short observation time—only 1 year (21). As an integrative risk prediction score derived from eight functional, histological, and immunological prognostic factors, in 2020 the U.S. Food and Drug Administration (FDA) also provided information to support the qualification of iBox as a reasonably likely surrogate endpoint (RLSE) in clinical trials evaluating immunosuppressive therapies in kidney transplantation (22). There is also another RLSE—the rate of decline of estimated glomerular filtration rate (eGFR) as a late post-transplant biomarker, that has been deemed acceptable by the FDA for use in a rare condition (chronic antibody-mediated rejection), however this biomarker remains to be validated for general use across clinical trials (5). Finally the Chronic Allograft Damage Index (CADI) adopts a sum score of six histopathological lesions in transplanted kidneys associated with graft function (23). CADI has been useful in clinical decision-making, by providing information on extent of chronic injury in the kidney allograft (23).

Finding accurate predictors depends on the immunological response, which can be highly variable due to immunosuppression therapies, comorbidities, and lifestyle factors. Transferring surrogate markers to new “surroundings” is also challenging as the predictive performance may not be the same and cross-validations may be necessary. For example, biomarkers evaluated in calcineurin inhibitor (CNI) based immunosuppression may not necessarily be valid in non-CNI protocols. The cost of immunosuppressive drugs and availability of follow-up visits also differ significantly across healthcare systems. Keeping these differences in mind will improve and ensure the comparability of treatment outcomes across geographies and treatment situations (24).

### Opportunities of Real-World Evidence

Both RWD and RWE refer to patient related data not collected through a RCT (25). “The diverse patient population, as well as broad scope of RWD sources makes it easier to generalize long-term outcomes and risks of a treatment compared to RCT results” (25). Additionally, discontinuation rates from regular follow-up in the transplant centres, captured by registries that may be statutory or otherwise mandatory, are much lower and ensure long-term continuity of data in studies that typically have less inclusion/exclusion criteria and are less invasive.

Innovative clinical trial designs, such as those using external comparators (ECs), harness the power of RWD derived from patients treated in RW settings (26). ECs, also sometimes referred to as “synthetic control data,” are used to provide context to a single arm study where it would be impractical or unethical to design the study with a placebo or active comparator arm (27). EC studies have different approaches in utilizing RWD for contextualization of trial data, and to supplement single arm trials. ECs can be used independently, for further contextualization while having a control arm in an RCT, or to supplement a control arm in an RCT (28, 29). ECs sourced from RW settings reflect the SoC, and whilst finding these control cohorts can be challenging and resource intensive, they provide context to the benefits and risks observed in single arm studies, and can provide insight into RW patient experiences. Furthermore, EC designs are likely to shorten time frames to regulatory submissions and lessen operational risks, and are increasingly used by regulators and government payers in difficult-to-recruit areas (30). Credible RWD needs to be of high quality, obtained from relevant sources, cleaned, harmonized, and—if needed—linked to additional data sources to fill in information gaps and include relevant endpoints to be fit for purpose (26). Within kidney transplant research, RWD could drive the conduct of pragmatic trials, EC studies, or the build of registries that can be used for nested trials^3^. Nonetheless, it is necessary to assess fitness for use of RWD by undergoing feasibility assessments before pursuing the study design.

The potential of RWE was seen in research by Friends of Cancer Research, where several RW clinical endpoints in patients with advanced non-small cell lung cancer treated with immune checkpoint inhibitors were compared to results of RCTs (29). Similar approaches are also in broader initiatives, notably the RCT Duplicate Initiative building an empirical evidence base through large-scale replication of RCTs (31). These pioneering projects ascertain the benefits of using RWD for extension studies and demonstrate the potential of ECs in future trial designs that study long-term outcomes to evaluate novel therapies.

There are two examples of kidney transplant studies, which followed a similar approach to an EC using extension studies (32, 33). The first compared rabbit antithymocyte globulin and basiliximab in kidney transplantation (32). To obtain 5-years follow-up data, patient trial records were matched with records in the Organ Procurement and Transplantation Network database for birth date, transplant date, sex, and transplant centre (32). This method allowed for extended follow-up, whilst also reducing costs of observation compared to prospective designs (32).

The second clinical trial is the tricontinental mycophenolate mofetil (MMF) kidney transplantation extension study, which initially recruited 503 patients that received a deceased donor kidney, and were randomized in equal groups to receive azathioprine (AZA) or MMF in combination with cyclosporine and steroids (11, 33). With 15 years of matched follow-up data from the Australia and New Zealand Dialysis and Transplant Registry, the study concluded little superiority of MMF over AZA (33). Linking the RCT to registries for long-term follow-up decreased biases compared against biases from purely observational designs (33).

RWE is increasingly required by regulators to demonstrate generalizable comparative insights, notably for: market authorization applications, line extension and post-authorization safety studies etc, (29). The non-invasive nature of RWD presents opportunities to assess long-term treatment outcomes using a combination of a properly designed clinical trials and registry outcomes data (17, 34).

Furthermore, RWD can be used to support the validation and test the predictive nature of short-term surrogate endpoints, as clinically meaningful surrogate endpoints that are predictive of final outcomes can be used and are needed for shorter term studies as well. Once such surrogate endpoints are validated, they could be used in clinical trials or other RW study designs. Specific transplant data (e.g., histology, immunology, and treatment) should be considered for consistent inclusion across registries, for example from diagnostic databases and biobanks, to expedite validation requirements.

### Identification of Most Relevant Real-Word Data Sources and Challenges in Generating Evidence From Them

The potential advantages of using RWD must outweigh concerns of quality and consistency (35). Not many existing registries capture sufficiently complete follow-up data for kidney transplant, which is a limitation of the RWE approach. Whilst some sources allow for nationwide assessments (e.g., cause of death), more consistent inclusion of surrogate endpoints, and biopsies, across follow-up periods are still needed to ascertain the cause of graft loss.

**FIGURE 1:**
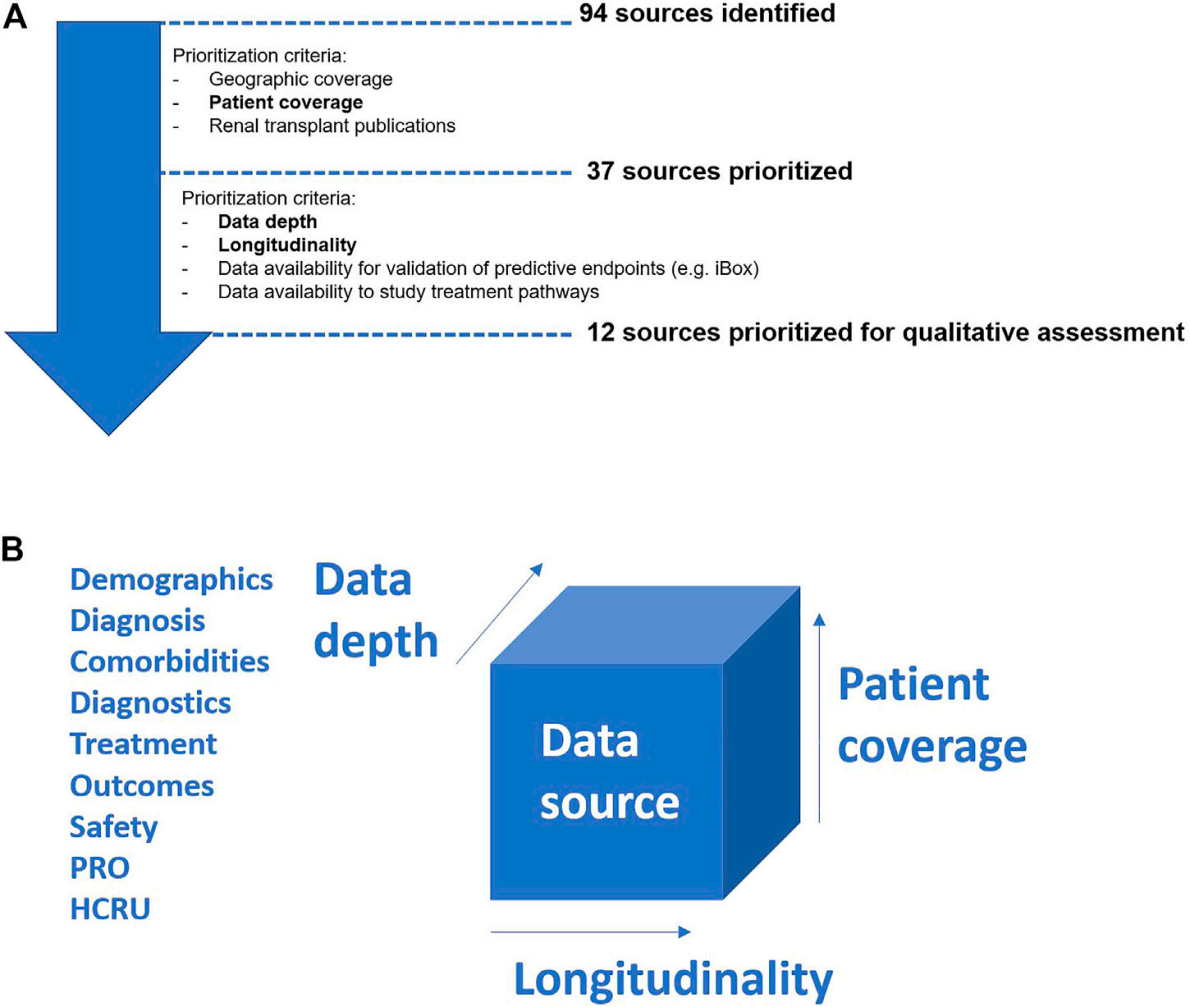
**(A)** Data source assessment process flow. Note: Bold terms refer to criteria employed by Framework 1b for assessing data sources. **(B)** Framework for assessing data sources. HCRU, health care resource utilization; PRO, patient reported outcomes. Note: In order to be suitable, data sources need to have both clinical depth, relevant patient numbers and a longitudinal capture that allows for the assessment of long-term outcomes.

**FIGURE 2:**
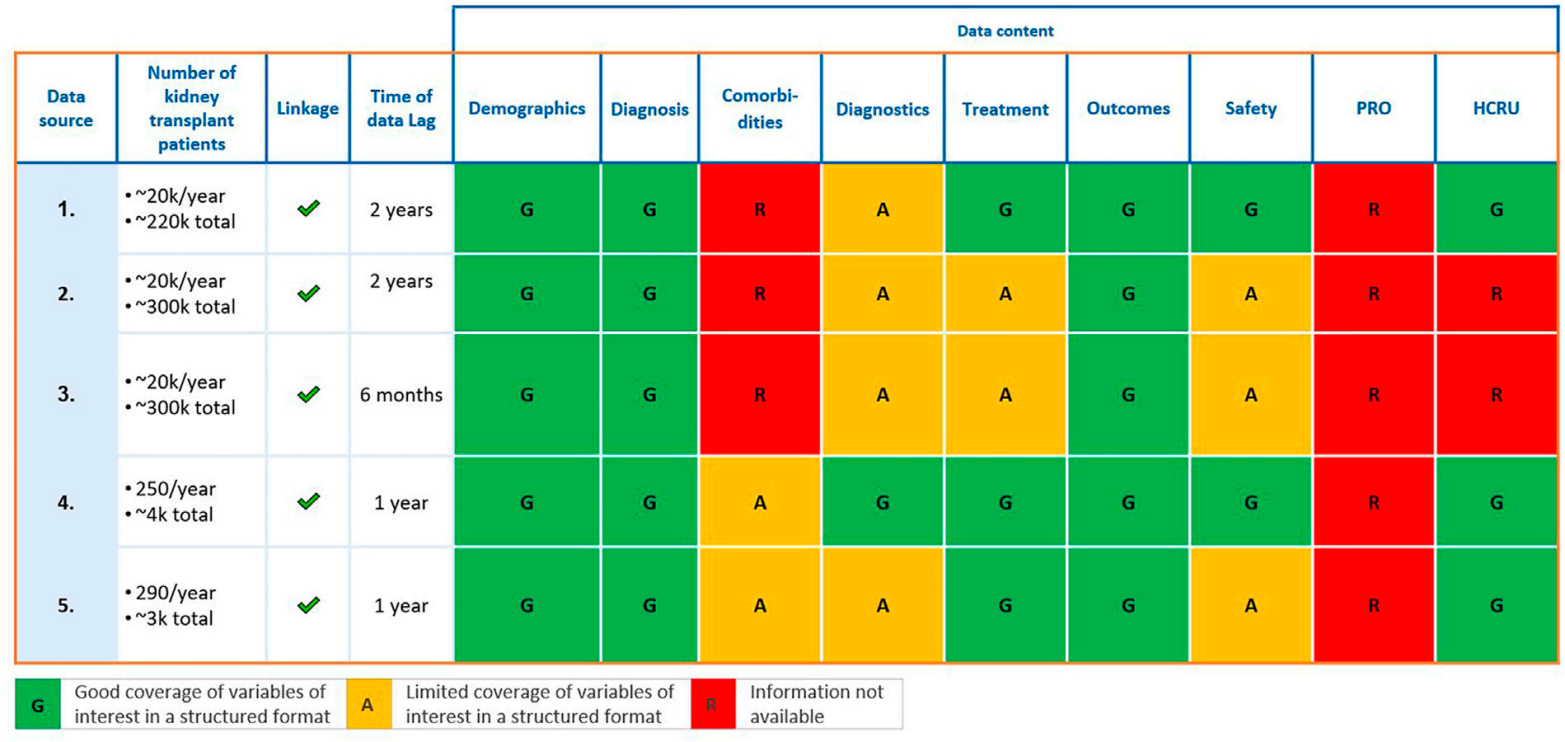
Five data sources qualitatively assessed. HCRU, health care resource utilization; PRO, patient reported outcomes.

A global literature search assessment was conducted by the authors in 2020, using a standard methodology described in Ekman et al., (36), to identify the most relevant RWD sources to assess treatment patterns, the clinical manifestations of AEs and validate predictive surrogate endpoints (e.g., iBox) in kidney transplantation (5). The search identified 94 RWD sources worldwide that had published research in English between 2010–2019, of which 37 were prioritized for in depth desk research based on publication record, patient and geographic coverage (Figure 1A). Further literature assessments for classification of data characteristics and follow-up found only 12 sources as preliminarily suitable for long-term assessments, of which five were qualitatively assessed during respective interviews with data source owners. Qualitative assessments aimed to determine database content, such as availability of variables, as well as research experience and ways of working (Figure 2) (36).

Whilst the five sources fully or partly met data requirements to assess treatment patterns, burden of disease, and validated predictive surrogate endpoints (e.g., iBox), they represent less than 10% of the kidney transplant sources identified. Hence, the assessment concluded that few kidney transplant RWD sources routinely capture data needed to derive predictive markers (e.g., tissue biopsy data for graft assessments) in greater clinical depth (Figure 1B) (36). Enhanced collaborations may alleviate the resource burden in order to produce and maintain long-term data, yet technical and semantic interoperability are required to overcome barriers that arise when harmonizing different sources (e.g., data standards, storage requirements, data handling procedures) (35). Failing to do so limits data utility, as seen in during the ADAPTABLE trial: divergence in data collection across facilities, and the “incomplete capture of past procedures and differences in classification of data,” limited comparison of doses of aspirin for prevention of hospitalization for myocardial infarction (35).

Identifying outcomes available across many sources, standardizing and enhancing data collection, will improve cross-source comparability to generate robust assessments. For example, more consistent glomerular filtration rate (GFR) measures would support definition of relevant surrogate endpoints for graft loss, and whilst this would likely require a shift from eGFR to standardized measured GFR assessments, this may be feasible with capillary samples and mathematical models. Ensuring such data breadth and completeness requires common definitions and sufficient time to implement changes that enforce required data quality.

Lastly, technology innovations such as Natural Language Processing^4^ and federated data models^5^, can support the building of larger cohorts with deeper structured data (37, 38). Such approaches enable rapid and consistent assessments across data depth, coverage, and temporality of capture. Federated data models utilizing clinical data repositories, and public-private partnerships, such as the Observational Medical Outcomes Partnership (OMOP), Patient-Centered Outcomes Research Network (PCORnet) serve as examples of international standards for data linkage and sharing. However, the practical considerations when using federated data models, such as ensuring linkage of disparate data sources, warrant caution (31). Use of RWD cohorts in innovative trial designs need to be aligned to prospective single arm trials with regards to population characteristics and definitions of data collected (28). Thus, to maximise the utility of harmonization by robust linkage and comparability, registries should more proactively develop common data modes to enable future research (39). This should be preferably done with the support from scientific transplant societies and consensus workshops and statements.

Several practical challenges exist in implementing large multinational registries with enough granularity and validated contemporary data for RWE studies. First, such a resource would be costly, and would require innovative design to start and maintain such a registry. Some examples exist however, where regulatory authorities are involved together with the industry, in funding and initiating a wide network of RWD, such as the EU-wide DARWIN (40). Another example of a private-public partnership project is the Transplant Therapeutics Consortium, including the different transplantation societies, FDA, and the industry (41). The inclusion of clinicians and clinical researchers as owners and curators of the datasets is vital for these types of joint efforts to be successful.

Another major hurdle for registry collaboration comes from ownership of data and data sharing policies, especially within the EU with the current General Data Protection Regulations (GDPR). Although GDPR should be EU-wide, individual countries have adopted very different policies for defining concepts of data transfer, making international collaboration sometimes challenging. One possible solution to this problem could be federated data models, described above, which allow for the generation of cohorts from different datasets without requiring data to leave.

## Conclusion

Sub-optimal long-term graft survival highlights the need for novel therapies and ways to demonstrate their long-term benefit-risk ratio for patients. Demonstrating superiority of novel therapies is unlikely in conventional RCT’s due to the financial and logistical burden of long-term follow-up. However, innovative designs have the potential to facilitate improved longitudinal transplant research by harnessing RWD sources to demonstrate both effectiveness and safety of treatment in a non-invasive, effective, and affordable way. Nonetheless, for innovative designs to bring more value to patients, a common understanding, definition, and agreement on surrogate endpoints predictive of final outcomes in kidney transplantation is required. For this to be possible, harmonization among registries via the alignment of definitions is crucial to improve the comparability and wealth of usable data across clinical practice, RCTs and registries.

The authors recognise that efforts are needed to strengthen the RWD infrastructure, thus also encourage developing studies of sub-populations and non-immunological aspects, as we believe these can demonstrate short and long-term benefits in situations where it may be methodologically hard to demonstrate superiority versus SoC in the general transplant population. Nonetheless, registry collaboration and data harmonization are considered key steps in demonstrating long-term beneficial outcomes of new therapies in kidney transplant patients (Table 1). Finally, clinicians, researchers and data owners are encouraged to explore multi-country collaborative studies that leverage registries, uptake of technology innovations, as well as the use of federated access and linkage from trials to RWE.

**TABLE 1:** Conclusions and Recommendations by the scientific forum.

Conclusions and Recommendations by the scientific panel
RWD^a^ sources, in combination with properly designed clinical trials, offer an effective and affordable way to assess long-term transplant outcomes. The FDA released guidance for industry to be used: “RWD: Assessing Electronic Health Records and Medical Claims Data To Support Regulatory Decision-Making for Drug and Biological Products” (42)
To enhance the use and impact of RWE^b^, registry collaborations and multi-country collaborative studies alike should work towards consistent selection of surrogate endpoints for increased comparability
Data harmonization that broadens patient coverage and extends follow-up should enable RWD to support the validation and test the predictive nature of short-term endpoints. Cross-source comparability assessments prior to harmonization are recommended for effective use of RWD [35]
Comparing data from different sources is possible even when pooling is difficult by leveraging technology innovations, including the use of federated models. Such approaches enable rapid and consistent assessments across data depth, coverage, and temporality of capture
Emerging innovative clinical trial designs that utilize RWD to complement trial data can provide additional benefits and shorten time frames to regulatory submissions. They require close alignment with regards to population characteristics and the definition of data collected

FDA: U.S., Food and Drug Administration; RWD, real-world data; RWE, real-word evidence.

a RWD: data relating to patient health status and/or the delivery of health care, not collected though clinical trials, but rather routinely collected from a variety of sources (electronic health records, claims and billing activities, product and disease registries, patient-generated data including in home-use settings, data gathered from other sources that can inform on health status such as mobile devices) (8).

b RWE, is the clinical evidence regarding the usage and potential benefits or risks of a medical product derived from the analysis of RWD (8).

## Data Availability

The raw data supporting the conclusion of this article will be made available by the authors, without undue reservation, provided applicable data protection regulations are complied with. The datasets presented in this article are not readily available because of applicable legislation protecting personal data. Requests to access the datasets should be directed to the corresponding author.

## Footnotes

1 RWD are the data relating to patient health status and/or the delivery of health care, not collected though clinical trials, but rather routinely collected from a variety of sources (electronic health records, claims and billing activities, product and disease registries, patient-generated data including in home-use settings, data gathered from other sources that can inform on health status such as mobile devices) (8).

2 RWE is the clinical evidence regarding the usage and potential benefits or risks of a medical product derived from the analysis of RWD (8).

3 Trials recruiting study subjects from a larger established study population whose characteristics are known

4 “a branch of artificial intelligence that helps computers understand, interpret and manipulate human language” (37).

5 “Data federation is an aspect of data virtualization where the data stored in a heterogeneous set of autonomous data stores are made accessible to data consumers as one integrated data store by using on-demand data integration” (38).

## Abbreviations

AE, Adverse event; AZA, azathioprine; CADI, Chronic Allograft Damage Index; CNI, calcineurin inhibitor; EC, External comparator; eGFR, estimated glomerular filtration rate; FDA, U.S. Food and Drug Administration; GDPR, General Data Protection Regulation; GFR, glomerular filtration rate; MMF, mycophenolate mofetil; OMOP, Observational Medical Outcomes Partnership; PCORnet, Patient-Centered Outcomes Research Network; RCT, Randomized controlled trial; RLSE, reasonably likely surrogate endpoint; RW, Real-world; RWD, Real-world data; RWE, Real-world evidence; SoC, Standard of care; SONG, Standardised Outcomes in Nephrology.
